# Drastic transformation of visceral adipose tissue and peripheral CD4 T cells in obesity

**DOI:** 10.3389/fimmu.2022.1044737

**Published:** 2023-01-04

**Authors:** Kohsuke Shirakawa, Motoaki Sano

**Affiliations:** Department of Cardiology, Keio University School of Medicine, Shinjuku-ku, Tokyo, Japan

**Keywords:** CD4 T cells, obesity, adipose tissue, osteopontin, immunosenescence

## Abstract

Obesity has a pronounced effect on the immune response in systemic organs that results in not only insulin resistance but also altered immune responses to infectious diseases and malignant tumors. Obesity-associated microenvironmental changes alter transcriptional expression and metabolism in T cells, leading to alterations in T-cell differentiation, proliferation, function, and survival. Adipokines, cytokines, and lipids derived from obese visceral adipose tissue (VAT) may also contribute to the systemic T-cell phenotype, resulting in obesity-specific pathogenesis. VAT T cells, which have multiple roles in regulating homeostasis and energy utilization and defending against pathogens, are most susceptible to obesity. In particular, many studies have shown that CD4 T cells are deeply involved in the homeostasis of VAT endocrine and metabolic functions and in obesity-related chronic inflammation. In obesity, macrophages and adipocytes in VAT function as antigen-presenting cells and contribute to the obesity-specific CD4 T-cell response by inducing CD4 T-cell proliferation and differentiation into inflammatory effectors *via* interactions between major histocompatibility complex class II and T-cell receptors. When obesity persists, prolonged stimulation by leptin and circulating free fatty acids, repetitive antigen stimulation, activating stress responses, and hypoxia induce exhaustion of CD4 T cells in VAT. T-cell exhaustion is characterized by restricted effector function, persistent expression of inhibitory receptors, and a transcriptional state distinct from functional effector and memory T cells. Moreover, obesity causes thymic regression, which may result in homeostatic proliferation of obesity-specific T-cell subsets due to changes in T-cell metabolism and gene expression in VAT. In addition to causing T-cell exhaustion, obesity also accelerates cellular senescence of CD4 T cells. Senescent CD4 T cells secrete osteopontin, which causes further VAT inflammation. The obesity-associated transformation of CD4 T cells remains a negative legacy even after weight loss, causing treatment resistance of obesity-related conditions. This review discusses the marked transformation of CD4 T cells in VAT and systemic organs as a consequence of obesity-related microenvironmental changes.

## 1 Introduction

The start of the obesity epidemic in the US in the early 1970s led to much research on adipose tissue, which regulates metabolic and nutritional homeostasis (1). Then, in the mid-1990s, Hotamisligil et al. proposed the intriguing hypothesis that immune cells are activated in response to a state of excess energy, which induces inflammation and, consequently, insulin resistance (2). Compared with subcutaneous adipose tissue, visceral adipose tissue (VAT) is composed of more diverse cell populations and is highly vascularized and contains numerous sympathetic and sensory nerves. Furthermore, during the development of obesity, a more complex diversity of immune cells arises in VAT than in subcutaneous adipose tissue. Since 2003, when Weisberg et al. and Xu et al. independently reported that macrophages accumulate in obese VAT, macrophages—which express high levels of inflammatory cytokines—have been considered the central players in VAT inflammation. However, subsequent studies revealed that VAT inflammation involves a greater variety of immune cells than previously thought and that not only the innate but also the acquired immune system is activated (3, 4). Obese VAT has a higher proportion of CD4 T cells, which have been recognized as central regulators of chronic VAT inflammation. Even in the lean state, VAT CD4 T cells continue to be weakly activated against an autoantigen specific to VAT. Interestingly, a much larger amount of regulatory T cells (Tregs) are present in VAT than in secondary lymphoid tissues, indicating that local immunometabolic homeostasis is maintained within VAT by enhanced immune tolerance (5).

During the development of obesity, strong activation of effector T cells initiates VAT inflammation (6). Furthermore, in chronic obesity, acute inflammation transitions to chronic inflammation and T cells become dysfunctional (because of T-cell exhaustion and senescence) (7–11). Once obesity has formed, the memory of obesity is imprinted on T cells and does not disappear with weight loss (12, 13); for this reason, weight rebound after weight loss induces more inflammation in the VAT than was present before weight loss (13).

VAT dynamically functions as not only a lipid storage organ but also an endocrine organ that produces a variety of soluble mediators, such as adipokines, cytokines, and lipids. The drastic changes in the VAT microenvironment in obesity affect the phenotype of not only VAT T cells but also systemic ones.

In this review, we summarize what is known about how T-cell activation, differentiation, and function change in response to obesity-associated factors.

## 2 Regulatory mechanisms of T-cell activity

In general, T-cell activation (survival, proliferation, differentiation, and functional enhancement) requires three positive signals: a T-cell receptor (TCR) signal, co-stimulation signal (CD28 signal), and cytokine signal. The TCR-mediated signal primarily activates the ZAP70-PLCγ pathway; the co-stimulation signal, the phosphatidylinositol-3-kinase-protein kinase B pathway; and the cytokine signal, the Janus family of kinases-signal transducer and activator of transcription (JAK-STAT) pathway (14). To suppress excessive responses, T cells are equipped with brakes on each of these three signals. TCR-mediated signaling is suppressed by programmed cell death 1 (PD-1) (15); PD-1 has an immunoreceptor tyrosine-based inhibitory motif, and upon stimulation by the ligands PD-L1 and PD-L2, it recruits Src homology 2 domain-containing protein tyrosine phosphatase 1, which dephosphorylates and inactivates ZAP7 (16, 17). Co-stimulation is suppressed by cytotoxic T-lymphocyte associated protein 4 (CTLA4), which has an extracellular domain similar to that of costimulatory receptor CD28 and competes with CD28 for binding to its ligand CD80/86; CTLA4 has higher affinity for CD80/86 than CD28 does, but it does not generate a signal (18) and thus physically blocks CD28-mediated signaling (19). And the cytokine signaling JAK-STAT pathway is suppressed by the suppressor of cytokine signaling family of molecules (20).

In addition to known T cell responses, obesity-associated insulin resistance and changes in adipokine secretion profiles all affect T-cell activation, function, and survival, not only in VAT but also in systemic organs (21). Obesity-associated adipocyte hypertrophy and hyperplasia cause VAT microenvironment remodeling, including impaired angiogenesis, deposition of extracellular matrix protein, and hypoxia-induced pyroptosis (22), which may result in changes in T-cell phenotype Obese VAT cells also regulate adipose T-cell activity through antigen presentation and co-stimulatory or co-inhibitory receptor signaling. Obesity-associated disruption of T-cell homeostasis may contribute to the development of an inflammatory state, which is followed by disruption of tissue homeostasis.

## 3 T-cell activation and suppression

The finding that T cells with a restricted TCR repertoire accumulate in the VAT of diet-induced obese (DIO) mice (23) suggests that in obesity, T cells recognize some kind of antigen. Antigen-presenting cells process an antigen and present it to T cells in the form of an antigen-peptide major histocompatibility complex (MHC). The MHC is recognized by the TCR on the T cell, and the T cell is activated. In addition, antigen-presenting cells highly express co-stimulatory molecules, which pair with co-stimulator receptors on the T-cell surface to modulate T-cell activation (24). Activation of T cells by antigen-presenting cells also plays an important role in triggering the VAT inflammation induced by a high-fat diet (HFD). In VAT T cells, MHC class II (MHCII)-T cell receptor interaction upregulates the expression of inflammatory Th1 marker genes, including *Tbx21* and *Ifng*. Deletion of MHCII, which plays a role in presenting antigen-derived peptides to CD4 T cells, reduces VAT CD4 T helper type 1 (Th1) cell activity and macrophage accumulation within VAT (23, 25–27).

In VAT, besides the classical antigen-presenting cells, i.e., dendritic cells (DCs) (28), macrophages (27, 29), and B cells (30), adipocytes (26) also play an important role in the formation of T-cell responses as antigen-presenting cells (31). Adipocytes express MHCII molecules and the costimulatory molecules CD80/86 and thus act as antigen-presenting cells and promote CD4 T cell activation (26).Interestingly, in obesity, adipocytes have higher expression levels of MHCII molecules and costimulatory molecules (25).The above findings indicate that during the progression of obesity, antigens presented on MHCII molecules induce T-cell proliferation and differentiation into specific subclasses of inflammatory effectors and that this process is the basis for the initiation and persistence of inflammation in VAT. Knowledge about obesity-related antigens could potentially lead to the development of vaccines and treatments to prevent chronic inflammation of VAT, but unfortunately, such antigens have not yet been identified.

Immune checkpoint molecules maintain immune homeostasis by suppressing self-immune responses and excessive activation of T cells. Co-inhibitory receptors such as CTLA-4 and PD-1 are co-expressed on effector T cells and are involved in immune response homeostasis (19). T-cell exhaustion is characterized by restricted effector function, persistent expression of inhibitory receptors, and a transcriptional state distinct from functional effector and memory T cells. In general, PD-1 is expressed in T cells in response to most immune challenges; however, it is rapidly downregulated during the acute phase of the immune response, allowing for normal immune responses (15). On the other hand, PD-1 expression remains high in chronically stimulated antigen-specific T cells, so the immune response to additional stimulation is impaired (32–35).

T-cell exhaustion has also been identified in VAT in mice and humans with obesity (7, 9, 36).

In fact, in HFD-induced obese mice and patients with type 2 diabetes, adipose T cells are less able than lean adipose T cells to produce cytokines such as IL-2 and IFN-γ (9). HFD-induced obesity increases the PD-1 expression level in adipose T cells, and T cells with high PD-1 expression are a subset of T cells that acquire the exhaustion phenotype (7–9). Various obesity-associated environmental factors lead to heterogeneous T-cell exhaustion profiles, suggesting that obesity may not promote classical T-cell exhaustion.The transient elevation in the expression of immune checkpoint molecules during the priming process, in which native T cells are sensitized and activated by antigen-presenting cells, not only inhibits excessive activation but is also involved in determining the polarity of the effector T cells (37, 38). PD-L1 is expressed in large amounts on DCs in VAT of HFD-fed mice. DC-specific PD-L1 deficiency shifts the polarity of T cells in the VAT of HFD-fed mice toward Th1, exacerbating weight gain and abnormal glucose metabolism (39). PD-L1 expression on DCs is an important factor in suppression of Th1, Th17, and cytotoxic T cells in antitumor responses and autoimmune diseases (40, 41) and appears to play a similar role in the pathogenesis of obesity.

Upregulation of PD-L1 in human adipose tissue is positively correlated with body mass index, but not with type 2 diabetes (42). On the other hand, a negative correlation was reported between body mass index and PD-L1 expression (43). The pathology of human obesity is highly heterogeneous, with multifactorial contributions by dietary quantity and quality, physical inactivity, and genetic factors. Therefore, despite the common phenotype of accumulation of adipose tissue, the number and qualitative changes of immune cells involved in chronic inflammation of VAT are thought also to be diverse. These differences may stem from the different roles of immune cells: They are involved in the initiation, amplification, and/or suppression of chronic inflammation of VAT, and their roles differ greatly depending on the phase of obesity. The role of PD-1/PD-L1 signaling in human VAT T cells remains unclear and requires further elucidation.

## 4 T-cell metabolism

Differentiation of CD4 T cells into functional subsets is supported by complex metabolic programs (44). Th1, Th17, and Th2 effector cells generate energy by aerobic glycolysis rather than oxidative phosphorylation, whereas Tregs rely on fatty acid oxidation-fueled oxidative phosphorylation rather than glycolysis (45).

Obesity-related alterations in environmental signaling in VAT alter cellular metabolism and contribute to obesity-specific T-cell responses (46, 47). However, it is difficult to measure the metabolic state of VAT T cells in mice because of the complexity of the isolation process, so splenic T cell metabolism has mainly been studied.

Glucose uptake and oxygen consumption are increased in splenic CD4 T cells from obese mice compared with those from lean mice (46). Furthermore, β3-adrenergic receptor stimulation mimics T-cell metabolism in DIO mice and reduces expression of the mitochondrial-localized chaperone protein disulfide bond A oxidoreductase like protein (DsbA-L) in T cells. Although alterations in mitochondrial respiration are an important mechanism controlling cytokine production, loss of DsbA-L in T cells reduces mitochondrial oxidative phosphorylation capacity in both CD4 and CD8 T cells. Mice with T-cell–specific knockout of DsbA-L have reduced IFN-γ–producing Th1 cells in brown adipocytes, enhanced brown adipocyte thermogenic signaling, and less obesity and insulin resistance when fed a HFD (48). Thus, the obesity-related changes in T cell metabolism greatly contribute to the pathogenesis of obesity, including insulin resistance; however, many aspects of the relationship between metabolism and function specific to VAT T cells remain unclear.

## 5 Substances that affect T cells in VAT, thymus and periphery

Adipocytes secrete a variety of bioactive substances, collectively referred to as “adipokines.” Changes in adipokines associated with the progression of obesity affect T-cell proliferation, differentiation, and function (49). Adipocyte-derived lipids also affect T-cell phenotypes in obesity (**Table 1**).

**Table 1:** Effects of humoral factors on CD4 T cells in obesity.

Humoral factors	Effect on CD4 T cells	References
Leptin	T-cell differentiation in the thymus	(50–52)
	Induction of Glut1 on effector T cells	(50)
	Promotion of Th17-cell differentiation	(53–57)
	Promotion of differentiation of IFN-γ–producing VAT Th1 cells	(26)
	Induction of T-cell exhaustion *via* STAT3 signaling	(8)
Adiponectin	Suppression of T-cell proliferation and cytokine production and promotion of apoptosis	(58–60)
	Suppression of MHC2, CD80, and CD86 on DCs	(21)
	Upregulation of PD-L1 on DCs and suppression of Th1-cell differentiation	(61)
	Inhibition of glycolysis pathway in Th1 and Th17 cells	(62)
Fatty acids	Differentiation of IFN-γ–producing effector T cells	(63)

DCs, dendritic cells; IFN-γ, interferon gamma; PD-L1, programmed death-ligand 1; STAT3, Signal Transducer and Activator of Transcription 3; Th1, T helper 1; VAT, visceral adipose tissue.

### 5.1 Leptin

Leptin stimulates the satiety center in the hypothalamus. As body fat mass increases, adipocytes produce more leptin, and the serum leptin concentration rises. In normal-weight individuals, the higher serum leptin concentration stimulates the satiety center, which suppresses eating behavior, allowing body fat mass to return to its base level. In the periphery, leptin promotes fatty acid oxidation and glucose uptake in skeletal muscle (64).

Leptin has been reported to have various physiological activities in both normal weight and obese conditions. It also acts on the immune system by promoting T-cell formation by the thymus. Conversely, leptin deficiency results in thymic atrophy and decreased numbers of circulating T cells (50). Administration of leptin to young leptin mutant (ob/ob) and normal mice increases CD4 single-positive thymocytes in the thymus and CD4 T cells in the periphery (51). The long-chain leptin receptor ObRb is expressed on double-negative, double-positive, and CD4 single-positive thymocyte subsets, but not on CD8 single-positive thymocytes (52). Among other things, leptin may promote differentiation from double-positive into CD4 single-positive cells (52).

In obesity, thymus function is reduced despite hyperleptinemia (65). Individuals with obesity are known to have high blood levels of leptin, but their appetite is not suppressed and they are in a leptin-resistant state. The question whether the leptin resistance observed in the hypothalamus is also observed in the thymus or whether other factors, such as adipogenesis of thymic tissue, are involved in obesity-related thymic hypofunction needs further clarification.

Leptin is also involved in the differentiation and proliferation of CD4 T cells in the periphery: TCR stimulation upregulates the expression of leptin receptors on T cells (66), which require leptin for metabolic reprogramming in which activated effector T cells upregulate the glucose transporter Glut1 and enhance the glycolytic system (50). Leptin also promotes differentiation of T cells into Th17 cells *via* T-cell–like leptin receptors (53–57).

In VAT, leptin gene expression in adipocytes begins to increase within 1 week after HFD loading, indicating that leptin may be an initiator of the adipose inflammatory cascade. Leptin promotes differentiation of VAT T cells into Th1 cells and secretion of IFN-γ (26). On the other hand, ob/ob and db/db mice also develop VAT inflammation and severe insulin resistance, suggesting that humoral factors other than leptin may contribute to the initiation of VAT inflammation (67). In addition, in the pathogenesis of chronic obesity, leptin signaling contributes to T-cell exhaustion by activating homeostatic STAT3 signaling and thus inducing PD-1 expression (8).

In VAT, in which adipocytes are the major constituent cells, T cells may be exposed to high concentrations of leptin. In addition, the degree and duration of obesity, which determine the amount of exposure to leptin, greatly affect T-cell phenotypic changes. Leptin is one of the initiators of VAT inflammation that promotes Th1 differentiation of VAT T cells, and long-term exposure to leptin induces immune exhaustion. Thus, leptin is a key molecule in obesity-induced T cell phenotypic changes.

### 5.2 Adiponectin

Adiponectin would be expected to prevent obesity and obesity-related diseases by promoting insulin sensitivity and fatty acid oxidation and exerting anti-inflammatory effects (68). However, serum levels of adiponectin are negatively correlated with obesity, and obesity decreases adiponectin receptor expression (68).

In general, most T cells store the adiponectin receptors AdipoR1 and AdipoR2 in intracellular compartments. After antigen-specific stimulation, AdipoR1 and AdipoR2 are transported to the cell surface and expressed along with the receptor CTLA-A and other receptors. In various *in vivo* inflammatory models, adiponectin acts as a negative regulator of effector T cells by suppressing T-cell proliferation and cytokine production and promoting apoptosis (58–60).

Adiponectin also appears to affect the mechanism by which naïve T cells are activated by DCs, and adiponectin treatment reduces the expression of MHCII, CD80, and CD86 on DCs and suppresses production of IL-12p40 (21). Furthermore, PD-L1 expression is increased in adiponectin-treated DCs. Co-culture of adiponectin-treated DCs with allogeneic T cells *in vitro* decreases T-cell proliferation and IL-2 production, and this phenomenon can be partially reversed by blocking the PD-1/PD-L1 pathway (61).

Although the question whether the obesity-related decrease in adiponectin releases the brake on the inflammatory response of VAT T cells remains unanswered, *in vitro* research suggests that adiponectin may suppress the inflammatory response of obesity-activated T cells. In fact, obesity enhances the glycolysis pathway in splenic Th1 and Th17 cells, and adiponectin inhibits glycolysis in both an AMPK-dependent and -independent manner, resulting in the amelioration of inflammation (62). In obese patients, adiponectin expression is reduced not only in adipose tissue but also in serum (69). The reduction in VAT-derived adiponectin may affect systemic immune function and contribute to the development of obesity-specific inflammatory conditions.

### 5.3 Free fatty acids

During the progression of obesity, lipids released from adipocytes are also involved in the enrichment of IFN-γ–producing CD4 T cells in VAT (63). The differentiation of TCR-stimulated IFN-γ–producing effector T cells is enhanced by co-culture with adipocytes (62). Among the soluble factors, fatty acids were shown to be the strongest modulators of differentiation into Th1 (63). Patients with obesity have high serum fatty acid concentrations, showing that the bias for differentiation of naïve T cells into IFN-γ–producing Th1 cells is not limited to VAT. Indeed, upon antigen presentation in secondary lymphoid tissues, T cells primed with excess saturated fatty acids have been shown to undergo biased differentiation into pro-inflammatory effector memories that tend to cluster in pro-inflammatory nonlymphoid tissues such as obese adipose tissue and atherosclerotic lesions (70).

Because VAT CD4 T cells appear to acquire unique characteristics that differ from those of CD4 T cells in other organs, including blood and spleen, it is unclear whether fatty acids have the same effects on CD4 T cells in VAT as they do on those in blood. However, VAT CD4 T cells are exposed to higher concentrations of fatty acids, which may result in higher production of IFN-γ and development of VAT inflammation.

## 6 Homeostatic and pathogenic role of CD4 T cells in VAT

From an immunological perspective, VAT is a unique environment. In the physiological (non-obese) state, T cells are kept weakly activated against a self-antigen specific to VAT, and Tregs that are highly reactive to self-antigens are also assembled. In this way, peripheral immune tolerance is enhanced to suppress inflammation in VAT and maintain immunometabolic homeostasis. During the development of obesity, the storage of excess fat leads to further enhancement of T-cell activation signals. However, in the chronic phase of obesity, T cells in VAT become dysfunctional. In this section, we describe how the immune-tolerant state of VAT is breached in obesity, how T-cell activation affects adipocyte function, and how T-cell transformation is involved in chronic VAT inflammation.

### 6.1 T cells under homeostatic conditions in a lean state

Endogenous tissue Tregs differentiate and mature in response to tissue-specific environmental signals and play an important role in organ homeostasis (5). VAT Tregs are functionally specialized tissue-resident cells that prevent obesity-associated inflammation and maintain insulin sensitivity and glucose tolerance (71). Under homeostatic conditions in a lean state, VAT has a large population of Tregs, and research showed that as non-obese mice age, Tregs accumulate in the VAT (72). Indeed, studies in mice found that at 20 to 30 weeks of age, VAT Tregs represent a surprisingly high percentage (50%-80%) of the CD4 T-cell compartment (73, 74). For reference, the proportion of Tregs in the CD4 T-cell compartment in spleen and lymphoid tissues is about 5% to 15%.

VAT is thought have such a high proportion of CD4 T cells because it requires Tregs for immunological homeostasis. Within VAT, T cells continue to be loosely activated by autoantigens in adipose tissue. Tissue-resident Tregs play an important role in preventing the activation of effector T cells and the accompanying dysfunction of adipocytes and in maintaining systemic insulin sensitivity.

There are two types of Tregs: Tregs that are generated in the thymus (referred to as naturally occurring or thymic Tregs) and Tregs that differentiate from naïve CD4 T cells in the periphery (peripheral Tregs) (75).

The intestinal lamina propria has a large population of Tregs to prevent excessive immune responses to dietary components and intestinal bacteria; the population comprises peripheral Tregs, which increase locally depending on intestinal bacteria, and thymic Tregs (76). When all germs were experimentally removed from the intestinal mucosa, the population of Tregs in the intestinal mucosa decreased to the level seen in lymphoid tissues (61). Among intestinal bacteria, *Clostridium* species are known to be a strong inducer of peripheral Tregs (76). In VAT, most Tregs are thymic Tregs. CD4 T cells that undergo negative selection in the thymus and do not die express the lineage-determining transcription factor Foxp3 to become Tregs, which are self-recognizing and thus inherently more likely to invade self-tissue. Compared with Tregs in lymphoid tissue, VAT Tregs have a unique TCR repertoire that exhibits specific antigen recognition (77), meaning that VAT Tregs react to certain antigens specific to VAT.

Foxp3 and the signal-dependent transcription factor peroxisome proliferator-activated receptor gamma (PPAR-γ), as well as signaling by the cytokine IL-33 through the IL-33 receptor ST2, are important for the proliferation and functional maturation of Tregs in VAT (78). VAT Tregs express insulin receptors, which are rarely expressed on Tregs in lymphoid tissues, and highly express PPAR-γ and the ST2 receptor in an insulin signaling-dependent manner (79). VAT Tregs have transcripts driven by PPARγ that differ from lymphoid-organ and other nonlymphoid-tissue Treg populations (80). Mice lacking Treg-specific PPARγ have greatly reduced VAT Tregs.

In mice, the VAT Treg population was found to show sex differences and IL-33 was found to be particularly important for the maintenance of Tregs in adipose tissue of males (81). Under homeostatic conditions in a lean state, the Treg population is larger in male than in female mice. In female mice, adipose tissue inflammation is suppressed by estrogen, and the expression of inflammatory cytokines such as IL-6, C-C motif chemokine ligand 2, and IL-1b in VAT is more pronounced in males than in females (81). Consistent with this finding, male mice are more insulin resistant than females. In males, the environment of higher inflammation in the VAT causes more Tregs to be recruited from the spleen. Androgens regulate the differentiation of IL-33–producing stromal cell populations specific to VAT in males (81).

In male mice, VAT Tregs upregulate expression of the ST2 receptor in a manner dependent on transcription factor Blimp1, which is also highly expressed in males; the upregulated expression leads to local expansion of VAT Tregs in an IL-33 signaling-dependent manner (81).

VAT Tregs also enhance IL-10 production in a Blimp1-dependent manner. IL-10 produced by VAT Tregs suppresses not only effector T activity but also white adipose tissue browning (82). Because female mice have smaller VAT Treg populations, they have lower IL-10 levels and are thus more prone to white adipose tissue browning; lower IL-10 may also be one of the reasons why female mice are protected from white adipose tissue accumulation and glucose intolerance compared with age-matched male mice.

As obesity progresses, VAT Tregs suppress inflammation by regulating effector T cells, DCs, and macrophage activity directly or *via* IL-10 production (74). Tregs, which constantly express CD25 (also known as IL-2 receptor alpha chain), preferentially bind to IL-2 to inhibit proliferation of effector T cells (74). Tregs express the adhesion factors lymphocyte function-associated antigen-1 (LFA-1) and CTLA4, and the former enables them to strongly adhere to DCs (83). In addition, Tregs downregulate the expression of CD80/86 on DCs in both a CTLA-4- and LFA-1-dependent manner (83). IL-10 acts mainly on DCs and macrophages to suppress production of IL-12 and tumor necrosis factor alpha (TNFα) and the expression of CD86, MHCII, and CD40 by strongly activating STAT3 (84).

In severe obesity, the Treg cell population is reduced to 10% to 20% of the CD4 T-cell compartment (73, 74). One of the reported mechanisms for this decrease is the presence of ST2 soluble isoforms, which are secreted by adipocytes in obesity and function as decoy receptors for the ST2 receptor to weaken IL-33 signaling (85, 86).

The immune compartments of adipose tissue are markedly different in mice, humans, and primates: Lean male mice have a high proportion of Tregs in VAT, but the proportion is much lower in female mice, humans, and cynomolgus macaques (87). In healthy lean humans, the proportion of Tregs is significantly lower in VAT than in peripheral blood; however, OX40-expressing Tregs, which have potent suppressive activity and high proliferative capacity, are selectively distributed in VAT in all individuals and further increase with obesity. Upregulation of OX40 Tregs is thought to be a protective mechanism that suppresses excessive inflammation (88).

Although the actions of immune cells in the maintenance of VAT homeostasis appear to differ greatly between species, Tregs play an important role in regulating VAT inflammatory across species.

### 6.2 Early stage of obesity-induced inflammation in adipose tissues

The main players in VAT inflammation in obesity are macrophages. In the lean state, macrophages localize diffusely in the VAT, but in obesity, they explode in number and become intensively localized as crown-like structures, which comprise clusters of macrophages surrounding dead adipocytes (89). A wide variety of T cells modulate monocyte recruitment to the VAT and macrophage proliferation and differentiation in the crown-like structures (90).

CD8 T cells were first noticed as initiators of VAT inflammation. CD8 T-cell infiltration into VAT precedes accumulation of macrophages, and genetic deletion of CD8 T cells reduces VAT inflammation and ameliorates systemic insulin resistance. Adoptive transfer of CD8 T cells into CD8-deficient mice exacerbates VAT inflammation (91).

CD4 T cells also regulate VAT inflammation. Macrophage-driven inflammation is induced by the Th1 response. In VAT, this response is induced by a HFD. Th1 cells produce TNFα, which activates the vascular endothelium and promotes monocyte invasion, and IFN-γ, which induces polarization of proinflammatory M1 macrophages (92).

Thus, Th1 cells contribute to the recruitment of monocytes into the VAT and their differentiation into M1 macrophages (26). IFN-γ suppresses insulin signaling in mature adipocytes, which attenuates insulin-dependent glucose uptake and lipid storage, and also inhibits differentiation of pre-adipocytes to mature adipocytes (93). In sum, Th1 cells play a central role in the induction of early adipose inflammation and adipocyte dysfunction associated with a HFD. Th1 cells are elevated in VAT in patients with type 2 diabetes and correlate with obesity-induced inflammation and insulin resistance (94). In human adipocytes, IFN-γ sustains activation of the JAK/STAT1 pathway, attenuates lipid storage and insulin signaling, and suppresses differentiation (93). Indeed, obese IFN-γ knockout mice have smaller adipocytes and less accumulation of VAT inflammatory cells, resulting in improved insulin sensitivity (95).

In HFD-induced obese mice, the Th17 cell population in the spleen and circulating levels of IL-17 are increased in an IL-6–dependent manner, and IL-17 inhibits insulin signaling in hepatocytes and glucose uptake in skeletal muscle (96). IL-17 has a modulatory effect on adipocytes, inhibiting lipid uptake and glucose uptake by insulin and suppressing adipogenesis (97). Obese insulin-resistant patients have more Th17 cells in their abdominal subcutaneous visceral fat than insulin-sensitive obese patients (98). Furthermore, in obese and metabolically unhealthy individuals, Th17 cells infiltrate obese adipose tissue and Th17 cytokines promote TNF-α production and induce inflammation (99).

Th2 cytokines, such as IL-4 and IL-13, have complex effects in VAT, i.e., they do more than antagonize Th1 cytokines and exert anti-inflammatory effects. In healthy adipose tissues, metabolic homeostasis is maintained by local IL-4 secretion by VAT eosinophils and maintenance of alternatively activated macrophages. In the absence of eosinophils, the numbers of alternatively activated macrophages in VAT are greatly reduced (100). In obese humans, the number of Th2 cells is decreased in subcutaneous and visceral fat and peripheral blood and Th2 frequency is inversely correlated with insulin resistance and serum levels of C-reactive protein, a marker of systemic inflammation (101). Th2 cytokines are abundant in the crown-like structures of obese VAT, where macrophages actively proliferate in a manner dependent on the IL-4 receptor α-chain (IL-4Rα), a molecule essential for Th2 signaling. In these structures, IL-6 functions as a major driver of proliferation of VAT macrophages in obesity by upregulating IL-4Rα (102). The role of the Th2 cytokines IL-4 and IL-13 in VAT inflammation caused by a HFD load is supported by the finding that inflammation is reduced in mice with myeloid cell-specific knockout of IL-4Rα (103). Thus, in the obese environment, Th2 cytokines play an important role in the maintenance of fast-proliferating macrophages within the crown-like structures (102). In the physiological state, innate lymphoid cells (ILC2s) are important producers of type 2 cytokines, which are critical for maintenance of alternatively activated or M2-like adipose tissue macrophages and glucose homeostasis (104). In obese VAT, ILC2s regulates saturated fatty acid absorption, resulting in the amelioration of chronic inflammation (105); however, in obesity the number of ILC2s decreases in both mouse and humans (104). The sources and roles of type 2 cytokines appear to be significantly different between VAT homeostasis and obesity-induced exacerbation of chronic inflammation, and further research is needed.

### 6.3 Obesity-induced chronic inflammation in adipose tissues

Overeating activates the effector function of T cells in VAT and initiates inflammation of the tissue. However, the mechanism by which this inflammation becomes chronic is still being elucidated. Recent studies have revealed that T-cell dysfunction due to both T-cell exhaustion and aging is involved in the chronicity of VAT inflammation, and these two aspects are discussed below.

#### 6.3.1 T-cell exhaustion

As a result of chronic inflammation, obese T cells exhibit an exhausted phenotype that includes long-term antigen-stimulation, stress responses, and hypoxia (106). Given that a significant proportion of VAT CD4 T cells are not recruited from the circulation and proliferate *in situ* (107), T-cell exhaustion can be expected to significantly interfere with antigen-specific and memory responses. Soluble factors from the obese stromal vascular fraction inhibit activation of VAT T cells, suggesting that the microenvironment of obese VAT may trigger T-cell exhaustion (9). In mice, chronic HFD intake attenuates the inflammatory capacity of effector T cells in VAT such that they lose their ability to respond to TCR-specific stimulation (9). Strikingly, VAT T-cell dysfunction has been suggested to occur early, i.e., before macrophage infiltration into the VAT (9).

VAT T cells from DIO mice fed a HFD for 18 weeks fail to upregulate CD25 or secrete T cell effector cytokines such as IFN-γ and IL-2 (9). CD25 is required for formation of TCR-stimulated, high-affinity IL-2 receptors. The same is true for VAT T cells in humans with obesity. On the other hand, the ability of Tregs to suppress effector T cells is reduced by downregulation of CD25.

The dysfunction of VAT T cells in DIO mice involves persistent antigen presentation from antigen-presenting cells. Although many T cells in the VAT of DIO mice express high levels of PD-1, blocking PD-1 in the VAT is not able to restore the ability to produce cytokines upon TCR stimulation (9).

In addition to persistent antigen presentation by antigen-presenting cells, some soluble factors in obese VAT are involved in T-cell dysfunction. One candidate for soluble factors is leptin (see also section 5.2). The high levels of leptin associated with obesity lead to T-cell exhaustion by inducing PD-1 expression through strong activation of STAT3 (8, 108). However, adipose tissue T cells from db/db mice also cause T-cell exhaustion (9), suggesting that it involves other humoral factors besides leptin.

Obese VAT is in a state of insulin resistance, in which glucose uptake and fatty acid synthesis are suppressed in the presence of high insulin levels and, conversely, triglycerides are broken down and fatty acids and glycerol are released (109). Impairments in angiogenic capacity also occur in obese VAT. Inhibition of angiogenesis leads to rarefaction of capillaries, impaired proliferation of multipotent progenitor cells, and adipocyte hypertrophy (110). These changes in the environment surrounding T cells may affect the intracellular metabolic system and induce T-cell exhaustion.

Aerobic glycolysis is activated when TCRs are stimulated and suppression of the glycolytic system induces elevated PD-1 expression and T-cell exhaustion, which decreases IFN-γ production. One group proposed a mechanism by which, when the glycolytic system is suppressed, glyceraldehyde 3-phosphate dehydrogenase binds to the 3’UTR of IFN-γ mRNA and inhibits its translation, resulting in decreased IFN-γ production (111).

In the cancer microenvironment, glucose deprivation by cancer cells results in reduced T-cell glycolytic flux, decreased IFN-γ production, and increased PD-1 expression (111). *In vitro* co-culture experiments have shown that in the cancer microenvironment, metabolic constraints, rather than chronic antigen stimulation per se, may be responsible for T-cell exhaustion (112). In the obese environment, a reduction in the glycolytic flux of VAT T cells may also accelerate T-cell exhaustion.

PD-1 is one of the indicators of T-cell exhaustion, and inhibitors of the PD-1/PD-L1 axis, which restores T cells from exhaustion, have provided major clinical breakthroughs in cancer (16, 113–115). PD-1 expression on T cells is elevated in people with obesity (8). Highly exhausted T cells are susceptible to PD-1 blockade, and obesity is positively correlated with the efficacy of PD-1/PD-L1 inhibitors in patients and mice with cancer (8, 116, 117), together suggesting that obesity induces T-cell exhaustion.

Obesity also induces CD4 T-cell exhaustion in obese VAT, and exhausted CD4 T cells highly express PD-1 (7). However, the finding that PD-1 blockade does not improve VAT CD4 T-cell exhaustion indicates that VAT CD4 T cells may have acquired a unique exhaustion phenotype independent of PD-1/PD-L1 signaling (9). CD8 T cells also appear to acquire diverse exhaustion traits in obese VAT. VAT CD8 T cells from obese mice highly express T-cell exhaustion markers such as *Pdcd1*, *Tox*, *Entpd1*, *Tigit*, and *Lag3* and have an exhaustion profile similar to T cells chronically infected with Lymphocytic choriomeningitis virus (36). On the other hand, obese VAT CD8 T cells do not have increased immune checkpoints such as *TIM-3*, *LAG-3*, *TIGIT*, and *EB4* or exhaustion markers such as *TOX*, *TCF-7*, and *Eomes*; instead, VAT CD8 T cells upregulate the co-inhibitory receptor B and T Lymphocyte Attenuator (*Btla*), *Nlrc3*, and *Dicer1* genes, which suppress TCR signaling by a mechanism different from that of PD1 signaling (118).

Differences in T-cell exhaustion profiles in VAT may result from the intensity and duration of exposure to specific antigens associated with HFDs and obesity. Immunosuppressive signals of VAT are also considered to be a defense mechanism that suppresses excessive obesity-related inflammation of VAT. However, VAT is an important reservoir of immune cells in obesity, and exhaustion of VAT T cells may be disadvantageous in case of various pathologies, such as infectious diseases and cancer.

To sum up, in developing obesity, a HFD load causes acute inflammation by inducing excessive activation of VAT T cells. On the other hand, in the chronic phase of obesity, inhibitory signals suppress the normal VAT T cell response. The fact that chronic inflammation in VAT persists suggests that the presence of not only T-cell exhaustion but also cell-intrinsic functional changes in VAT T cells, which more actively sustain inflammation.

#### 6.3.2 Regression of the thymus and maintenance of T cells by homeostatic proliferation

TCR spectratyping analyses show that diet-induced obesity causes thymic regression and limits TCR diversity. Obesity due to melanocortin-4 receptor deficiency, the most common genetic cause of human obesity, also reduces T-cell repertoire diversity (65). A key factor in obesity-induced thymic regression is the conversion of thymic fibroblasts to adipocytes as a result of lipid accumulation (119). Adipocyte proliferation leads to an increase in leukemia inhibitory factor, oncostatin M, and IL-6, which inhibits thymic function and induces thymocyte apoptosis, resulting in a compromised pool of T-cell progenitor cells (120). After thymic degeneration, peripheral T cells are maintained by homeostatic proliferation. In humans, homeostatic proliferation of circulating CD4 T cells is accelerated in individuals with obesity (121).

#### 6.3.3 Mechanism of the repertoire restriction of T cells

VAT T cells have significantly less TCR diversity than splenic T cells, and obesity exacerbates this difference (23). Repeated antigen stimulation, the effects of antigen-presenting cells themselves, and homeostatic proliferation may contribute to the repertoire restriction of T cells.

After thymic degeneration, peripheral T cells are maintained by homeostatic proliferation, but a significant proportion of VAT CD4 T cells are not recruited from the circulation (107). Furthermore, VAT contains a huge pool of T cells in obesity (122). These findings suggest that to compensate for the decrease in VAT T cells, VAT T cells are activated and maintained by repeated antigen stimulation rather than by homeostatic proliferation. Studies showed HLA- and MHC2-mediated activation of T cells by antigen-presenting cells in obese humans and mice (25–27, 123, 124), suggesting that repeated stimulation with some kind of antigen may result in TCR repertoire restriction. Differences in the expression of various co-stimulatory and inhibitory receptors expressed on antigen-presenting cells also influence T-cell phenotypic changes (24–27, 29, 31), so antigen-presenting cells may also contribute to T-cell repertoire restriction.

On the other hand, a specific subset of T cells that emerges with obesity may be maintained through homeostatic proliferation because studies showed that during the physiological aging process, T cells are maintained through homeostatic proliferation triggered by homeostatic cytokines and MHC associated with self-peptides (123, 124) and we previously found that obesity accelerates T-cell senescence and that senescent T cells have similar characteristics to T cells created by homeostatic proliferation during aging (7). Although no studies have directly demonstrated homeostatic proliferation of T cells in VAT, we hypothesize that obesity-associated senescent T cells may be maintained by homeostatic proliferation in the same way as aging-associated senescent T cells.

#### 6.3.4 Obesity-induced T cell senescence

Cellular senescence is triggered by DNA damage, telomere dysfunction, inflammation, and metabolic dysfunction and is accompanied by irreversible cell cycle arrest and the acquisition of the senescence-associated secretory phenotype (125). Oxidative stress, inflammation, and repeated antigenic stimulation associated with obesity may induce shortening of telomere length and accelerate cellular aging (111, 126, 127). Obesity is also associated with leukocyte DNA methylation changes that can lead to immune dysfunction (128). However, the effects of long-term exposure to obesity on gene expression and metabolic status in T cells remain to be elucidated.

In mice fed a HFD, the absolute number of CD4 T cells per gram of VAT continues to increase as obesity progresses. One study attributed this increase in the absolute number of VAT CD4 T cells to an increase in antigen-stimulated activated CD44^hi^CD62L^lo^ cells (7). In this study, a unique population of CD44^hi^ CD62L^lo^ CD4 T cells that constitutively express CD153 and PD-1 was found to exhibit cellular senescence properties. A CD153^+^ PD-1^hi^ subset expressing T-bet, the master transcription factor of Th1, had high senescence-associated beta-galactosidase activity and were positive for the DNA damage marker γH2AX, indicating characteristics of cellular senescence. The authors concluded that CD153^+^ PD-1^hi^ subset continues to secrete abundant osteopontin (OPN) without PD-1–mediated negative signaling inhibition at the cost of normal function and causes VAT inflammation (7). In fact, OPN is elevated in the circulation of patients with obesity and enhances VAT inflammation, leading to the development of diabetes (129, 130).

OPN functions as a potent chemoattractant for macrophages. Chronic inflammation is characterized by macrophage retention, and OPN is a particularly important molecule in promoting macrophage migration and retention. OPN also regulates cytokine production by macrophages and acts on T cells to promote IL-12 production while inhibiting IL-10 production and promoting Th1 cell-mediated responses (131).

A portion of Th1 effector cells terminally differentiates into OPN-producing senescent T cells, and increased numbers of senescent T cells produce large amounts of OPN and maintain high levels of OPN in VAT (7). In turn, high OPN levels cause persistent accumulation and activation of macrophages and induce a Th1 response. These experimental results suggest that, at least in mice, VAT CD4 T cells that have deviated from immune checkpoint mechanisms and acquired a senescent phenotype continue to produce OPN independently of classical TCR stimulation and that this process is closely related to the maintenance of chronic inflammation in obesity. Recently, vaccination targeting CD153 was shown to reduce senescent cells in visceral fat of obese mice fed a HFD for 10 to 11 weeks and to improve VAT inflammation and insulin resistance (132). Senescent T cells were suggested as a potential therapeutic target for obesity-associated immunometabolic disorders.

CD153^+^ PD-1^hi^ CD4 T cells were originally identified as a characteristic T-cell subset that emerges with aging and were named senescence-associated T cells (124, 133). Noteworthy is that the same cells also appear in the VAT microenvironment in obesity and are involved in chronic local inflammation, making T cell senescence a common underlying pathology for both obesity-related and aging-related diseases.

VAT CD4 T cells play an important role in regulating inflammation and metabolism in obesity, but the underlying mechanisms are largely unknown. Recently, DIO was shown to increase p38 activity in VAT T cells and promote obesity-associated adipose tissue senescence. p38α, an essential subunit of p38, promotes T-cell glycolysis through a mechanistic target of rapamycin signaling, resulting in enhanced Th1 differentiation. T cell-specific deletion of p38α protected mice from HFD-induced obesity, fatty liver, adipose tissue inflammation, and insulin resistance (134).

**Figure 1:**
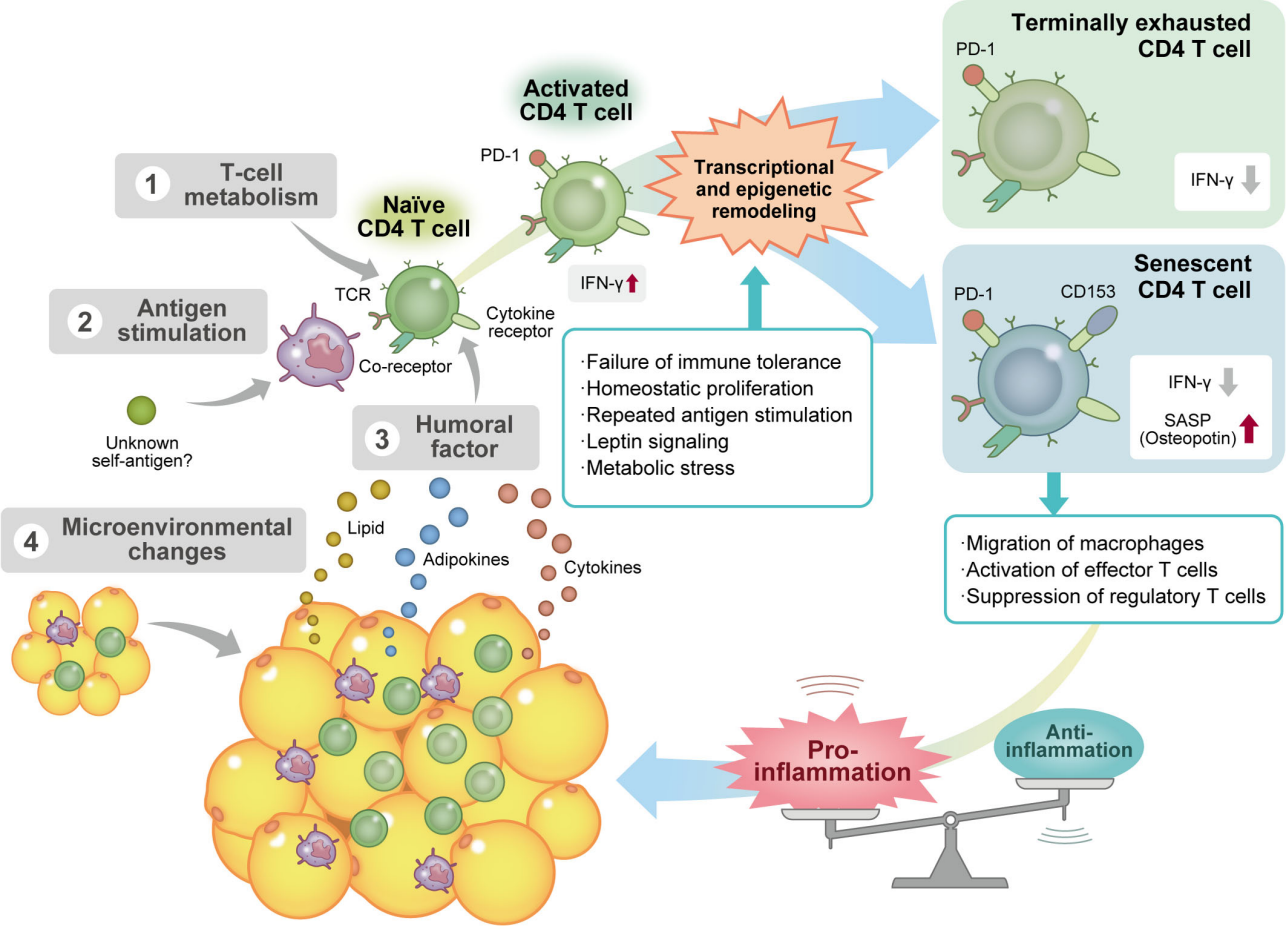
Obesity induces CD4 T-cell senescence *via* several mechanisms. Factors affecting the differentiation and maturation process of visceral adipose tissue (VAT) CD4 T cells in obesity include 1) alteration of T-cell metabolism; 2) repeated antigen stimulation by antigen-presenting cells, such as macrophages and dendritic cells; 3) various humoral factors, such as cytokines, adipokines, and lipids; and 4) changes in the VAT microenvironment. These stimuli activate T helper 1 (Th1) CD4 T cells and induce various types of transcriptional and epigenetic remodeling. Chronic activation of T cells induces immune exhaustion and reduces the secretion of proinflammatory cytokines, including interferon γ. On the other hand, some Th1 effector T cells deviate from the immune checkpoint mechanism and acquire a cellular senescence phenotype without anergy because of repeated antigenic stimulation. Senescent CD4 T cells that produce a large amount of osteopontin accelerate macrophage migration and effector T-cell activation and suppress regulatory T cell function, resulting in the maintenance of chronic VAT inflammation. Ifn-γ, interferon γ; PD-1, programmed cell death protein 1; SASP, senescence-associated secretory phenotype; TCR, T-cell receptor.

To sum up, the negative linkage between obesity-accelerating T-cell senescence and T-cell activation-accelerating VAT senescence plays an important role in the development of chronic VAT inflammation (**Figure 1**).

## 7 Negative legacy of obesity

Obesity can be corrected by weight loss. However, weight loss through caloric restriction and increased physical activity is not as easy as it sounds, and even if weight is lost, it may be regained quickly. Traditionally, bariatric surgery has been performed in severely obese patients who are refractory to medical therapy because of a combination of factors such as dietary environment and unique personality traits. With the recent launch of a glucagon-like peptide 1 receptor agonist and dual glucose-dependent insulinotropic polypeptide/glucagon-like peptide 1 receptor agonist, long-term maintenance of successful lost weight by medication has finally become feasible (135, 136). However, studies on the long-term effects of weight reduction on cardiovascular events and life expectancy in patients with obesity and type 2 diabetes have yielded conflicting results. A meta-analysis showed that weight reduction is associated with reduced mortality in unhealthy individuals with obesity (137), and bariatric surgery reduced the incidence of myocardial infarction in patients with obesity and type 2 diabetes over a mean follow-up period of 13.3 years (138). However, a prospective randomized study found that an intensive lifestyle intervention focused on weight reduction was not associated with reduced cardiovascular disease and mortality in adults with overweight or obesity and type 2 diabetes at almost 10 years of follow-up (139).

**Figure 2:**
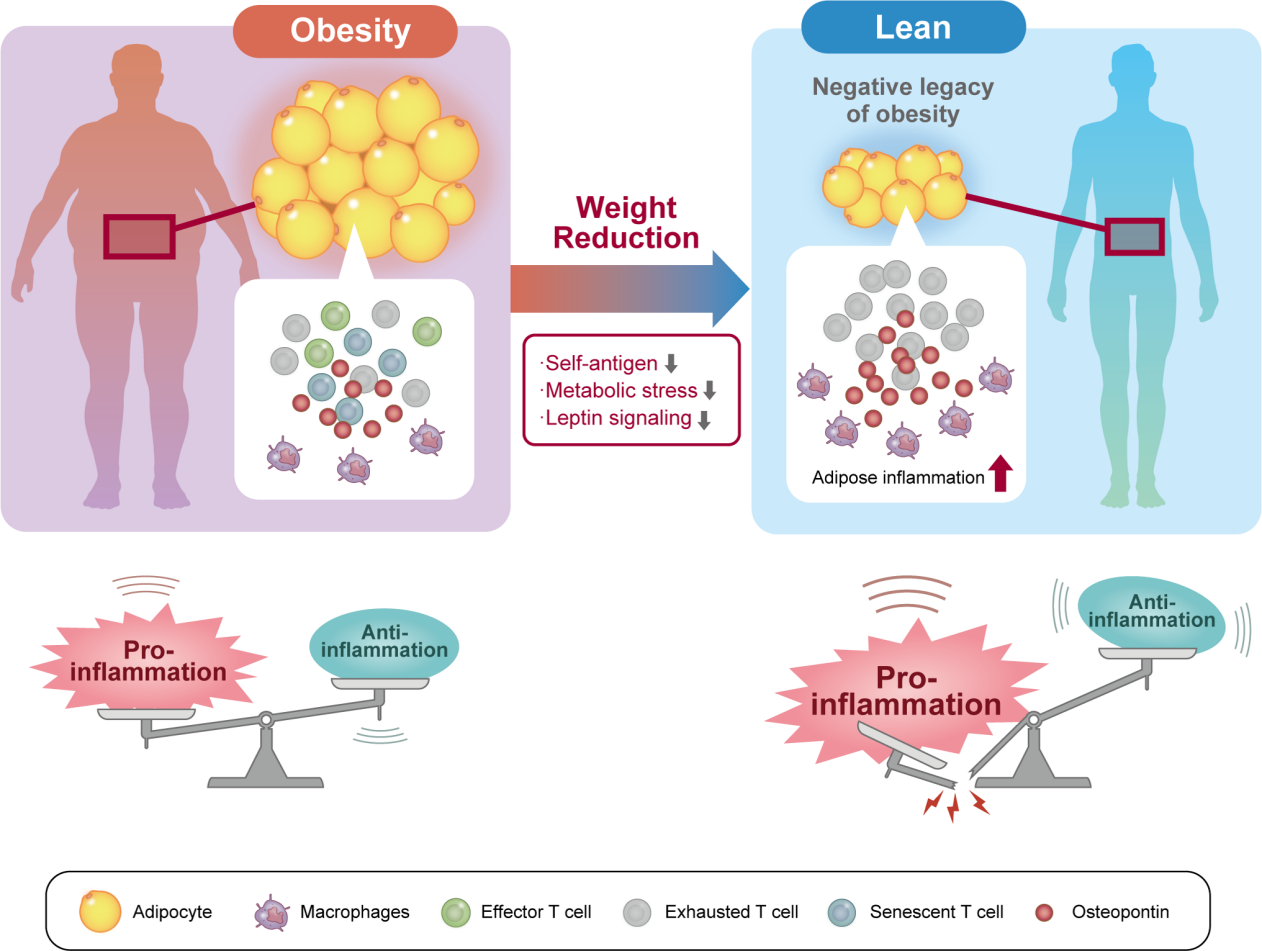
Senescent CD4 T cells are a negative legacy of obesity. In the pathophysiology of obesity, not only effector and exhausted T cells but also senescent T cells accumulate in visceral adipose tissue (VAT). VAT inflammation induced by these VAT T cells induces systemic insulin resistance and contributes to the pathogenesis of diabetes. Weight reduction reduces exposure to self-antigens, metabolic stress, and humoral factors in the VAT microenvironment. However, long-lived senescent CD4 T cells concentrate in VAT after weight loss and continue to secrete proinflammatory osteopontin. As a result, VAT inflammation persists after weight reduction and may represent a residual risk factor for cardiovascular disease.

The phenotypic changes in T cells upon weight reduction in obesity appear to be very complex. In obese mice, weight reduction induces the accumulation of CD4 T cells in VAT, resulting in the recruitment and retainment of pro-inflammatory macrophages in VAT despite normalization of body weight (13, 140). In mice with weight reduction after being switched from a HFD to a control diet, body weight and visceral fat decreased to the same level as lean mice fed the control diet; however, the VAT of the weight-loss mice showed dense infiltration of macrophages, which formed more crown-like structures than those in HFD-fed obese mice. Mechanistically, CD153^+^ PD-1^hi^ CD4 T cells are long-lived and not easily eliminated after weight loss, and the continued presence of senescent T cells is associated with the production of large amounts of OPN, creating a chronic inflammatory loop (**Figure 2**). Thus, senescent CD4 T cells are suggested to be a negative legacy effect of obesity (13).

Interestingly, in a weight gain-loss-regain model, mice with a history of obesity regain weight very quickly. Although the molecular mechanism for this obesity memory is unknown, CD4 effector T cells were shown to be involved (12). Furthermore, one study reported that glucose tolerance was worse in DIO weight-loss-regain mice than in normal DIO mice and that this metabolic dysfunction was associated with increased T-cell accumulation in VAT (36).

The above experimental results explain the body’s propensity to repeatedly gain and lose weight, which makes it difficult to maintain weight loss and increases the risk of developing diabetes. Noteworthy is that the background imprinting of an obesity-related immune phenotype is present in VAT.

## 8 Conclusions

VAT is not only a storage organ for lipids, but also an endocrine organ that regulates energy balance in the body by secreting various bioactive substances called adipokines, which are involved in food intake and insulin resistance. Dysregulation of adipokine function and production in obese VAT plays a major role in the development and progression of metabolic syndrome. Furthermore, it is becoming clear that various immunocompetent cells, including macrophages, increasingly infiltrate obese VAT and that the accompanying mild systemic chronic inflammatory response is the underlying pathogenesis of lifestyle-related diseases such as metabolic syndrome.

In this review, we sought to summarize the role of CD4 T cells in maintaining VAT homeostasis, inflammation caused by excessive fat accumulation in VAT, and the chronicity of this inflammation. In addition to TCR signals, co-stimulation signals, and cytokine signals from antigen-presenting cells, T cells undergo tissue-specific differentiation in response to organ- and disease-specific environmental signals.

To date, most of the findings on chronic inflammation of VAT associated with obesity are from analyses of DIO mice fed a HFD rich in saturated fatty acids. Therefore, future research needs to carefully examine the extent to which findings from DIO mice can be extrapolated to humans. Epidemiological evidence indicates that obese VAT increases the risk of developing cardiovascular disease. However, the mediators that affect remote organs *via* immunological changes in VAT remain unclear. In obesity, ectopic fat accumulates around the heart and blood vessels, and development of cardiovascular disease may be more strongly influenced by this neighboring ectopic fat. Obesity also affects systemic immune function. When considering the relationships between obesity and infectious diseases, cancer, and autoimmune diseases, the hypothesis is attractive that immunological abnormalities in obese VAT can also affect systemic immune function. In fact, some research suggests such an association, but proving a causal relationship remains a challenge for the future. Nevertheless, an understanding of the qualitative and quantitative changes of immune cells within the microenvironment in VAT will undoubtedly help to elucidate the mechanisms by which obesity causes various diseases, including lifestyle-related ones, *via* excessive local or systemic activation of the immune system. In the future, tailored therapeutic strategies based on an understanding of immune cell trait changes may contribute to improving the prognosis of obesity-related diseases.

## Author contributions

Both KS and MS contributed to conceptualization, writing (original draft preparation, review, and editing), and funding acquisition. All authors contributed to the article and approved the submitted version.
